# Effect of Electroacupuncture on ^99m^Tc-Sodium Pertechnetate Uptake and Extracellular Fluid Free Molecules in the Stomach in Acupoint ST36 and ST39

**DOI:** 10.1038/s41598-018-24835-9

**Published:** 2018-04-30

**Authors:** Rui Gao, Shan Gao, Jinteng Feng, Hongying Cui, Yanchao Cui, Junke Fu, Guangjian Zhang

**Affiliations:** 1 Department of Nuclear Medicine, The First Affiliated Hospital of Xian Jiaotong University, Xi’an, Shaanxi China 710061; 2 Department of Thoracic Surgery, The First Affiliated Hospital of Xian Jiaotong University, Xi’an, Shaanxi China 710061; 3 Department of Traditional Medicine, The First Affiliated Hospital of Xian Jiaotong University, Xi’an, Shaanxi China 710061

## Abstract

Electroacupuncture (EA) is a therapeutic modality in which the electrical stimulation is integrated with concepts of acupuncture to treat diseases. This study was designed to evaluate the connection between the electro-acupuncture induced increase in Na^99m^TcO4 uptake in the stomach wall, and the ionic molecule levels in the extracellular fluid in the acupoints. Wistar rats were treated by 2 or 100 Hz EA at Zusanli (ST 36) and Xiajuxu (ST 39) bilaterally for 60 minutes. The accumulation of Na^99m^TcO4 in the gastric wall and the free ions, including Ca^2+^, K^+^, Na^+^, and Cl^−^, in the acupoints were measured every 60 minutes. The radioactivity uptake in the stomach was significantly increased during EA, reaching peak at 180 minutes after the EA. The concentration of extracellular ions was also significantly increased during EA. The Ca^2+^ level continued to rise until 60 minutes after EA, then started to decrease at 120 minutes post-EA. The results suggest this up-regulatory effect of EA on gastric activity might be triggered by the increase of the extracellular ion levels, this effect lasts longer than stimulating the release of transmembrane Ca^2+^ flow alone. This might aid in providing a better understanding of the long-lasting effect claimed in acupuncture treatment.

## Introduction

Acupuncture, as an integral part of Traditional Chinese Medicine, has long been used to treat diseases^1^. Recent research suggested acupuncture may take effect by changing the cellular K^+^, Na^+^, and Ca^2+^ levels^2^. Experiments indicated the possibility of the biological modulation information of acupuncture encoded in the magnitude, frequency, and spatial organisation of the changes in the cellular-free Ca^2+^^3,4^. The latency and memory effects of Ca^2+^ oscillations that remained at 30 minutes or even 1.5 hours after the needle stimulation was turned off appeared to agree with the long-lasting healing claimed in acupuncture treatment^5^.

Electroacupuncture (EA) is a therapeutic modality in which the principles of electrical stimulation are integrated with traditional concepts of acupuncture^6^. Zusanli (ST 36) and Xiajuxu (ST 39), among others acupoints, are most extensively studied for their positive effects on various gastrointestinal disorders^7^. Although several categories of assessment methods were applied to investigate the modulation process on the gastric organs, the explanation of the mechanisms and effects of EA on those organs remains unclear^8^. Based on previous studies, we hypothesised that the EA-induced up-regulation of the stomach function, manifested by an increase in radiopharmaceutical ^99m^Tc-sodium-pertechnetate (Na^99m^TcO4) uptake surrounding the fundus of the stomach, might be induced by the ionic molecule levels changing in the acupoints on the stomach meridian^9^.

Microdialysis (MD) is a specific, local sampling method to collect free molecules of interest from the extracellular fluid^10^. It has been used widely in both animals and human subjects to investigate the changes experienced by extracellular molecules or medicine under certain physical or pathological conditions^11^. In our hypothesis-driven study, we set up an MD system for ionic molecule detection at the acupoints in rats undergoing EA, and checked the correlation between these free-molecule levels and the photon activity in the stomach. As far as we are aware, no reports are found to investigating this issue for acupuncture.

## Materials and Methods

### Animals

**Figure 1:**
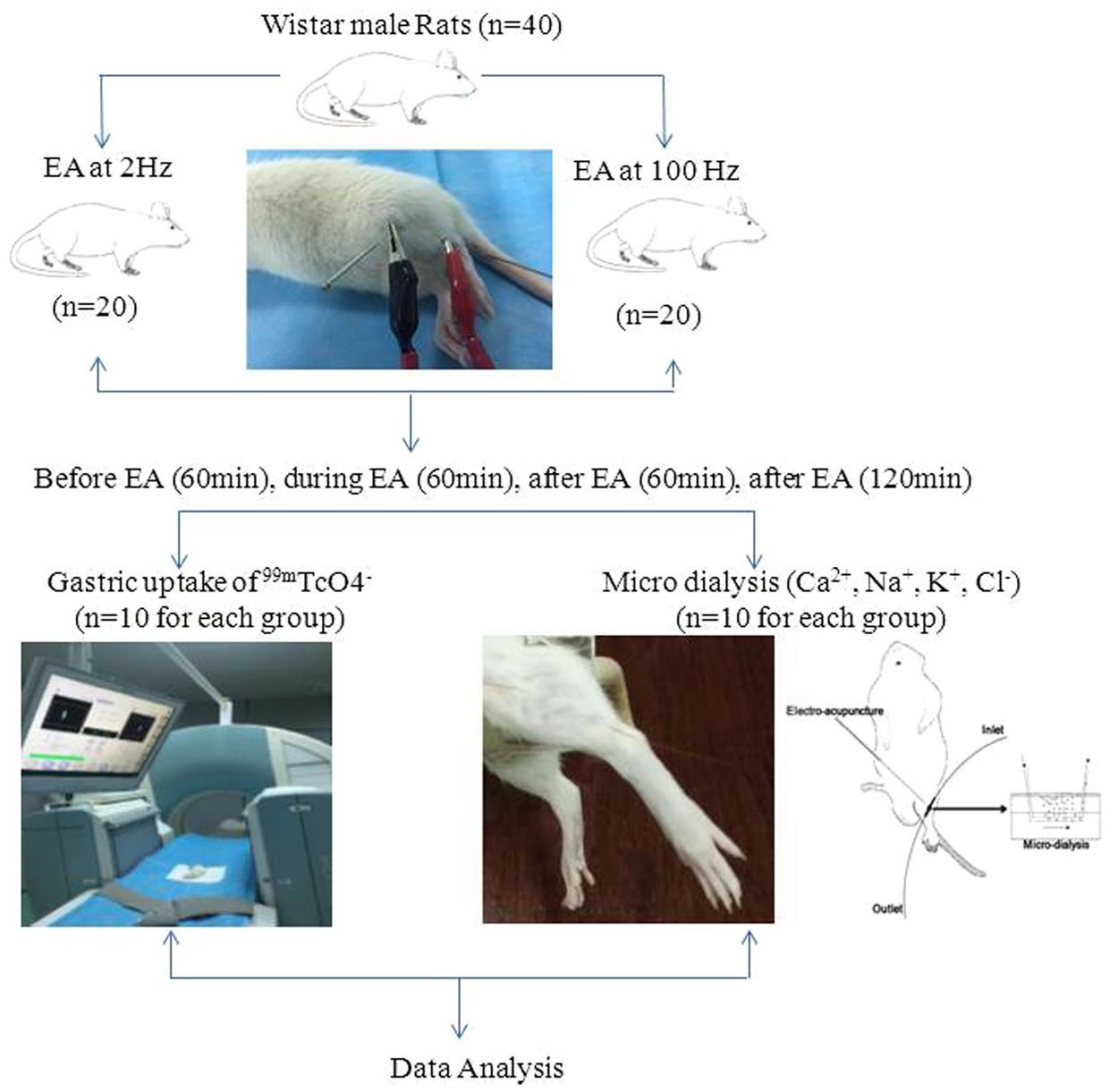
Flow chart of our experiment.

Sixty healthy male Wistar rats, weighing 180 to 250 g, aged 4 to 5 months, were used in this study. They were housed at a room temperature (22 ± 3 °C) under standard 12-h light/dark cycles (lights on at 07:00) with unlimited access to food and water, all rats were acclimatized for one week. The experiments were performed according to the guidelines of the Institutional Animal Care Committee of the University. All experiments were approved by the Ethical Committee of The First Affiliated Hospital of Xian Jiaotong University (Approval Dossier #: 2015-067). All efforts were made to minimize the number of animals used and their suffering. The rats were randomly chosen and divided into three groups (n = 20 each): G1, treated by 2Hz-EA at Zusanli (ST 36) and Xiajuxu (ST 39); G2, treated by 100 Hz-EA at ST 36 and ST 39 (Fig. 1). Half of the rats (n = 10 for each group) were assigned for SPECT/CT for evaluating gastric function, and the other half received microdialysis measurements.

### Electroacupuncture treatment

Rats were anaesthetized with an intraperitoneal injection of 20% Urethane at a dose of 5 mg/kg. Rats were placed in a ventral decubitus position. EA was applied at Zusanli (ST 36) and Xiajuxu (ST 39) bilaterally as previously described^12^. EA was given for a total of 60 minutes (1 mA stepwise to 3 mA, 0.2 ms duration, 2 or 100 Hz). Rats in the non-EA group were also anaesthetised in a similar fashion for 60 minutes as described^12^.

### Microdialysis system components, target molecule detection, and calculation

The CMA 30 Linear Microdialysis Probe (CMA Microdialysis, Stockholm, Sweden) to study the peripheral tissues (diameter 0.24 mm, length 21 cm, membrane length 10 mm) was used. The probe consists of inlet tubing, outlet tubing, and a middle part with a window where the membrane is located. The inlet of the probe was connected to a peristaltic pump (CMA Microdialysis, Stockholm, Sweden), which would push the tube with normal saline (NS) at a rate of 2.0 µL/min. Before sample collection, the membrane part of the probe was immersed in distilled water for 30 minutes. Then, the probe was immersed in the tissue around ST 36 (depth of about 3 mm), and the dialysate was collected in the outlet part every 60 minutes for the following 4 hours (i.e., before EA, during EA, 60 minutes after EA, and 120 min after EA). The dialysate was stored at −20 °C for further analysis.

We tested the target molecules, detecting efficiency of the probe with standard ion solutions. The MD probe was put into NS for 20 minutes for stabilisation and then was switched to standard ion solutions. The standard ion sampling was collected at 60-minute intervals. All diluted dialysates samples were analysed for Ca^2+^, Na^+^, K^+^, and Cl^−^ concentrations by LABOSPECT008 (Hitachi Japan). Dialysate ions were calculated as^13^:

$$\begin{array}{c}{\rm{The}}\,{\rm{target}}\,{\rm{molecules}}\,{\rm{detecting}}\,{\rm{efficiency}}={\rm{Measured}}\,{\rm{ion}}\,\mathrm{concentration}/\mathrm{Standard}\,{\rm{ion}}\,{\rm{solution}}\,{\rm{concentration}}\\ {\rm{Extracellular}}\,{\rm{ion}}\,{\rm{concentration}}={\rm{Detected}}\,{\rm{ion}}\,\mathrm{concentration}/\mathrm{Target}\,{\rm{molecules}}\,{\rm{detecting}}\,{\rm{efficiency}}\end{array}$$

### Na^99m^TcO4 imaging of the stomach wall

Na^99m^TcO4 SPECT/CT was performed 5 minutes after the injection of 7.4 MBq of ^99m^TcO4^−^ via the caudal vein. The scanners were dual-head gamma cameras, using low-energy high-resolution collimators and a 20% energy window centred on 140 keV. The DICOM image files of each rat were saved on optic discs and transferred to a Symbia Evo Excel workstation (Siemens Healthineers) for centralized reconstruction, reading, and analysis.

Na^99m^TcO4 SPECT/CT images were read and interpreted by the consensus of three experienced nuclear medicine physicians with reference to SPECT/CT fusion images. For semiquantitative analysis, gastric-to-background ratios of SPECT images were measured and calculated by the same person using a consistent standard^14^. The data are collected and compared with before EA, during EA, and after EA, to observe the adjusting effect of EA on gastric function.

### Statistical analysis

The data were analysed using SPSS 10.0 software (SPSS Inc. Chicago, Ill., USA). All results are expressed as the mean ± standard error (SEM). Two-way ANOVA and Duncan post hoc tests were performed to investigate the effects of electroacupuncture on the extracellular ions concentrations and on the gastric functions during each session, and a *p*-value < 0.05 was considered statistically significant.

### Availability of data and materials

The data sets used and/or analyzed during this study are available from thecorresponding author upon reasonable request and have been approved by the institutional committee on scientific research.

### Ethics approval

This study is approved by the ethics committee on biomedical study of the Xi’an Jiaotong University Health Science Center.

## Results

### The Ions levels at ST 36 increased significantly during EA

As reported previously, the target molecules detecting efficiency of the probe was evaluated with the standard solutions of Ca^2+^, K^+^, Na^+^, and Cl^−^ (Table 1).

**Table 1:** Target molecules detecting efficiency of the probe (mmol/L).

Ions	Standard solutions	Concentrations detected	Target molecules detecting efficiency
Ca^2+^	8.52 ± 0.08	5.74 ± 0.72	67.40%
Na^+^	40.90 ± 0.27	23.40 ± 0.70	57.21%
K^+^	25.21 ± 0.13	14.54 ± 1.75	57.68%
Cl^−^	26.42 ± 0.33	15.85 ± 0.76	61.22%

Stimulating ST 36 with 2Hz-EA increased the concentration of extracellular Ca^2+^ significantly during EA (*p* = 0.003 vs pre-EA). The Ca^2+^ level increased gradually and reached the peak plateau at 60 minutes after 2Hz-EA (*p* = 0.75 vs EA), then started to decrease at 120 minutes post-EA (*p* = 0.04 vs EA). The concentrations of extracellular Na^+^ and Cl^−^ were also significantly increased during EA as compared with pre-EA (*p* < 0.001 and *p* = 0.007, respectively). After EA, they started to decrease. Their levels decreased to 71.81 ± 15.09 mmol/L and 57.42 ± 14.30 mmol/L 60 minutes after 2Hz-EA (*p* = 0.09 and *p* = 0.07 vs. EA). The level of extracellular K^+^ showed similar changes, although the comparisons showed no significance (Table 2).

**Table 2:** The effect of EA at 2 and 100 Hz on the extracellular ion levels (mmol/L).

Ions	Hz	60 min before EA	60 min during EA	60 min after EA	120 min after EA
Ca^2+^	2	0.16 ± 0.11	0.36 ± 0.16*	0.40 ± 0.15	0.21 ± 0.11^$^
	100	0.18 ± 0.12	0.53 ± 0.15^#^	0.58 ± 0.16	0.42 ± 0.13
Na^+^	2	51.53 ± 9.35	78.43 ± 11.83*	71.81 ± 15.09	66.29 ± 12.84
	100	53.02 ± 8.54	85.62 ± 10.67^#^	80.94 ± 12.46	72.53 ± 11.37
K^+^	2	3.93 ± 1.41	4.46 ± 1.55	3.28 ± 1.30	3.14 ± 1.09^$^
	100	3.79 ± 0.99	5.27 ± 1.29^#^	4.96 ± 1.64	4.25 ± 1.28^¶^
Cl^−^	2	45.95 ± 10.04	61.11 ± 12.35*	57.42 ± 14.30	48.26 ± 13.06^$^
	100	43.64 ± 11.91	66.38 ± 13.40^#^	59.79 ± 13.62	50.56 ± 12.58

^*^60 min during 2 Hz EA *vs* 60 min before 2 Hz EA, *p* < 0.05; ^$^120 min after 2 Hz EA *vs* 60 min during 2 Hz EA, *p* < 0.05; ^#^60 min after 100 Hz EA *vs* 60 min during 100 Hz EA, *p* < 0.05; ^¶^120 min after 100 Hz EA *vs* 60 min during 100 Hz EA, *p* < 0.05; Two-way ANOVA followed by Duncan post hoc tests.

**Figure 2:**
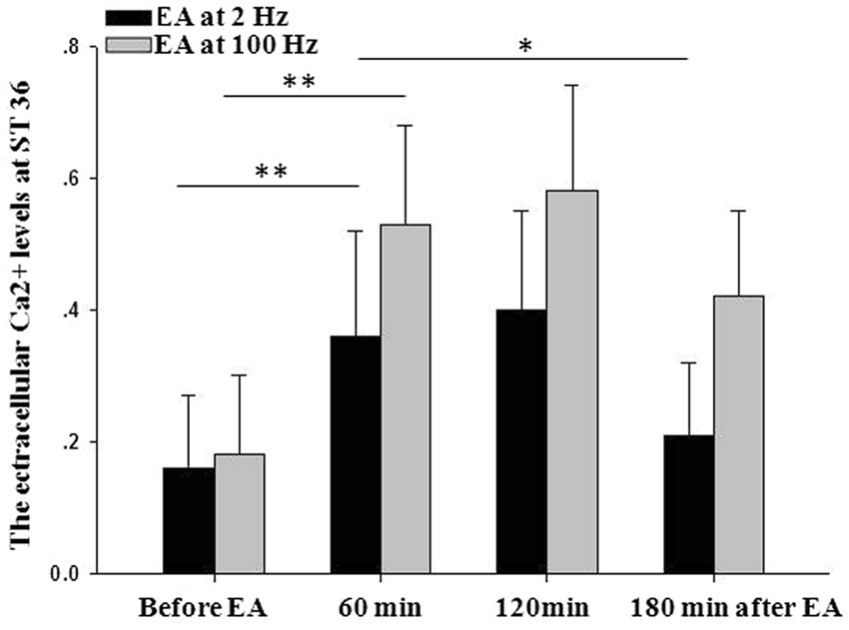
Extracellular Ca^2+^ levels at acupoint ST 36 under electroacupunture. The Ca^2+^ level at acupoint ST 36 increased significantly during 2Hz-EA (*p* = 0.003 vs. pre-EA). The Ca^2+^ level continued to increase till 60 minutes after EA, and then started to decrease in post-EA at 120 minutes (*p* = 0.04 vs. EA). Similar to the situation under 2 Hz, the extracellular Ca^2+^ concentrations also increased significantly under high frequency (100 Hz) EA, and the increase started to decrease 120 minutes after EA. The difference between low and high frequencies (2 *vs* 100 Hz) did not reach significance. Compared with extracellular Ca^2+^ levels during EA, *p < 0.05, **p < 0.01, Two-way ANOVA followed by Duncan post hoc tests.

Compared with the 2 Hz group, EA at 100 Hz also significantly enhanced the increase of the extracellular Ca^2+^, Na^+^, and K^+^ concentrations during EA, and the effect did not disappear 60 minutes after EA. However, no significant difference was found between the two groups (Table 2, Fig. 2).

### The radioactivity uptake in the stomach was significantly upregulated during EA

**Figure 3:**
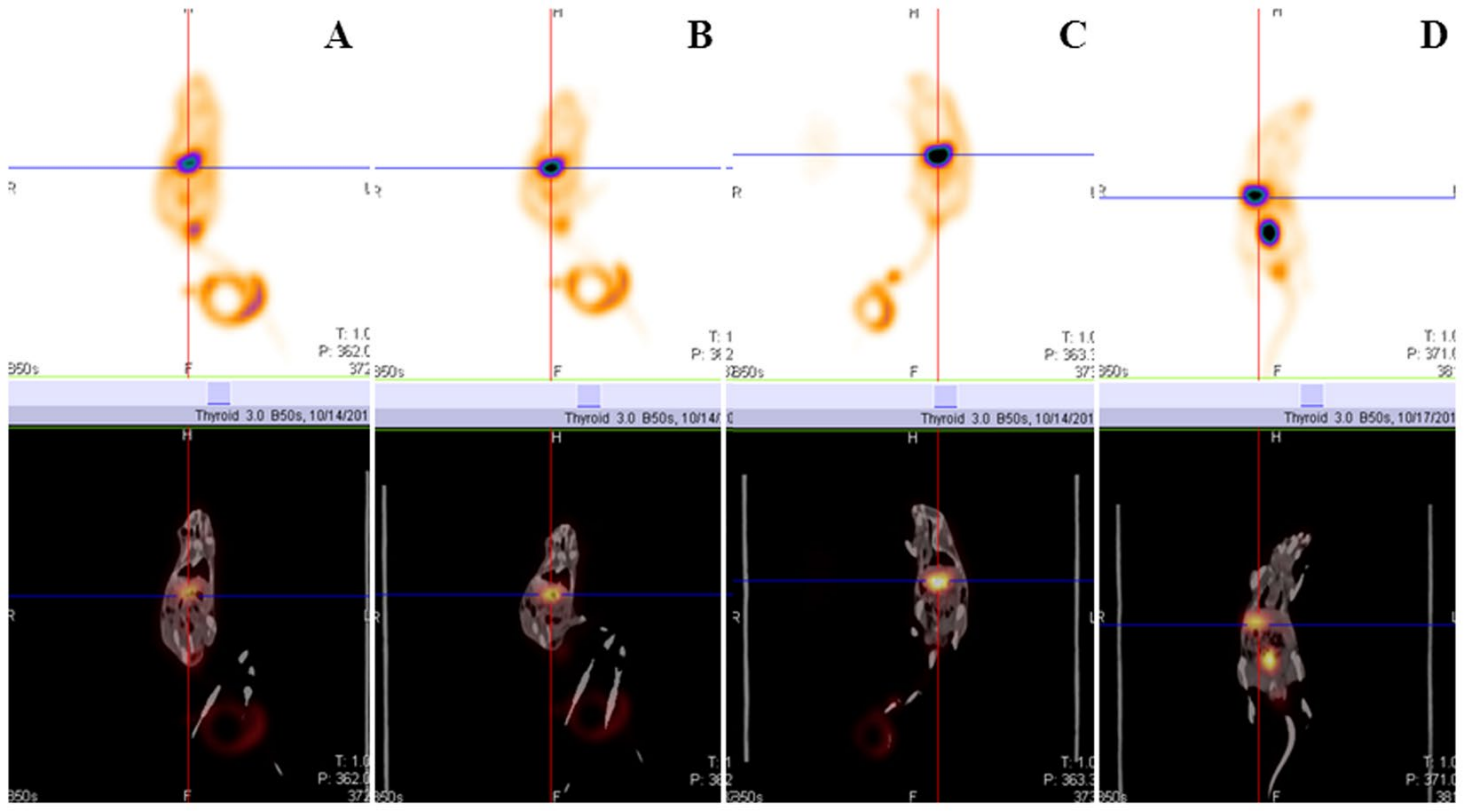
As compared with before EA group (**A**), the radioactivity uptake in the stomach was significantly increased during 2 Hz EA (**B**). The level of stomach Na^99m^TcO4 uptake did not reached the peak until 180 min after EA (**C**), and then started to decrease 240 min after EA (**D**).

Compared with before EA, the radioactivity uptake in the stomach was significantly increased during 2 Hz-EA (T/B ratio, 10.98 ± 0.75 mmol/L vs 8.96 ± 0.99 mmol/L). Interestingly, the up-regulation increased gradually (T/B ratio, 14.74 ± 0.46 mmol/L and 18.72 ± 0.84 mmol/L at 60 min 120 min after EA, respectively) and did not reach the peak until 180 minutes after the EA (T/B ratio 22.80 ± 1.40 mmol/L at 180 min after EA). Then the stomach uptake started to slowly decrease 3 hours after 2 Hz EA at ST 36 (Fig. 3).

**Figure 4:**
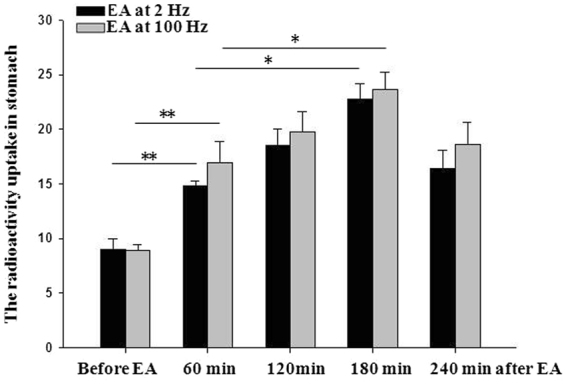
Compared with pre-EA, the radioactivity uptake in the stomach was significantly increased during 2 Hz-EA. The up-regulation escalated gradually and did not reach the peak until 180 minutes after the EA. The stomach uptake started to decrease 3 hours after 2 Hz EA at ST 36. 100 Hz-EA appeared to induce more radioactivity uptake than did 2 Hz EA in the stomach; however, no significance was reached in comparison of the two groups. Compared with radioactivity uptake in the stomach during EA, *p < 0.05, **p < 0.01, Two-way ANOVA followed by Duncan post hoc tests.

100 Hz EA appeared to induce more radioactivity uptake than did 2 Hz EA in the stomach; however, no significance was reached when comparing the two groups (Fig. 4).

## Discussion

Studies have shown functional gastrointestinal tract (GIT) disorders can be treated by acupuncture^1,2^. The most commonly used acupuncture points in treating GIT symptoms are Zusanli (ST 36) and Xiajuxu (ST 39)^1^. In traditional Chinese medicine, ST 36 and ST 39 are the points of the “Stomach Meridian of Foot-Yangming,” which is reported to be useful in treating GIT disorders such as stomach ache, abdominal pain and distension, constipation, diarrhoea, vomiting, dysentery, and indigestion^1,2^.

The unbound form of pertechnetate anion (Na^99m^TcO4) is distributed throughout the vasculature and interstitial fluids by a slow diffusion mechanism, and the concentration varies between organs^15^. In the gastrointestinal tract, Na^99m^TcO4 is actively concentrated mainly in the gastric mucosa^16^. The bioavailability of Na^99m^TcO4 in the gastric wall can be changed in many circumstances, including the patient’s health status, disease states, invasive medical procedures, and complementary therapies such as acupuncture^17^. Despite that the specificity of acupuncture is still questioned, the actions of acupuncture at ST 36 have been shown to significantly increase the radioactivity uptake of Na^99m^TcO4 in the stomach^9^. In the current study, we utilized the Single positron emission tomography (SPECT/CT) as main testing modality to observe the gastric function under electroacupuncture (EA) *in vivo*. (SPECT/CT) could reproduce anatomical and physiological imaging by mapping GIT changes *in vivo* in response to acupuncture stimuli^18^. Our results suggest the up-regulation effect of EA on gastric wall Na^99m^TcO4 uptake. Previous studies have suggested that EA could produce an excitatory effect on the gastrointestinal motility of the rat^9^. These ST 36 acupuncture effects may be correlated with our data, which suggested that 2 Hz-EA could obviously increase the uptake of Na^99m^TcO4 in the stomach, whereas no significant differences were found between the 100Hz-EA group and the control group.

Compared with simple acupuncture, EA is a therapy in which the principles of electrical stimulation are integrated with traditional acupuncture^6^. The two electrical frequencies 100 Hz (high frequency) and 2 Hz (low frequency) are commonly used for EA research^19^. Our results showed that EA at both frequencies could induce increases in gastric function, similar to previous studies. Tian *et al*. suggested that EA at both low and high frequencies could induce a therapeutic effect on obesity^20^. Tseng *et al*. showed that EA in either frequency could induce a decrease in glucose, an increase in lactate metabolites, and a reduction in lactate/glucose ratios^21^. Studies of EA in which ST 36 has undergone electrostimulation at 2 or 100 Hz have suggested that the mechanism underlying the effect might be that EA could lead to the regulation of internal organ systems as a network^22^. EA stimulation could lead to effects on gastric myoelectrical activity, brain-gut peptides responses in the gastric mucosa, and the vagal and splanchnic nerve responses on the activity of gastric motility and emptying^23^.

In recent years, more and more laboratory proof has accumulated that acupuncture can change the charge and potential of neurons, the concentrations of K^+^, Na^+^, Ca^2+^, and the content of neurotransmitters^3–5^. Among them, Ca^2+^ was supposed to play a key role^5^. As an important intracellular second messenger, Ca^2+^ participates in various physiological and biochemical processes of cells^24^. The proposed acoustic signaling paths, jing-luo, are distinctly and physically separated from the nervous system. This appears to be the missing “calcium” communication link proposed but not found in the frequency-encoded second messenger^25^. It has been suggested that calcium may be taken as the carrier of the biological modulation system, of which acupuncture has been implemented as a clinical application^26^. Moreover, because the intracellular Ca^2+^ concentration is controlled by the opening and closing of both voltage-dependent and receptor-dependent Ca^2+^ pathways, as well as by the Na^+^/Ca^2+^ exchanger and the activity of the Ca^2+^ pump, the concentrations of K^+^ and Na^+^ were reported to change with Ca^2+^ levels^27^.

The effects of acupuncture at the points of the Foot-Yangming meridian on gastric movement are related to the release of intracellular Ca^2+^ in the gastric smooth muscles^1^. The AM and FM schemes of calcium signaling in the acupuncture was proposed to cause cellular Ca^2+^ flux. The signal is broadcasted with a chevron-like pattern radiating from the needle like a radio antenna^28^. Our study showed that acupuncture at ST 36 and ST 39 could markedly enhance release of intracellular Ca^2+^ and, thus, significantly increased the extracellular Ca^2+^ levels. The extracellular Ca^2+^ level increase was correlated with gastric function upregulation. One potential testable hypothesis could be that the AM and FM schemes of calcium signaling were proposed based on the amplitude modulation of extracellular Ca^2+^ levels, and separated from the central nervous system, there might be a channel of cellular communications with calcium waves playing the role^3^. This proposed mechanism might explain our surprising finding that the increase of the gastric wall Na^99m^TcO4 uptake lasted longer than the enhancing of the release of intracellular Ca^2+^ to extracellular space observed in our study. Similar observations have been reported that Ca^2+^ oscillation remained at 30 minutes or even 1.5 hours after the needle stimulation was turned off^5^. The latency and memory effects appeared to agree with the long-lasting healing claimed in traditional acupuncture treatment^29^.

In this experimental model, the authors did not include the “sham acupuncture” group as an “inactive” control, since needling non-acupoints (any cutaneous location of the body surface) could produce unpredictable somatovisceral reflex responses, and therefore could complicate the interpretation of the results^30^. Our experimental finding is that the when EA was applied at non-acupoints, the coupling of the increase of Ca^2+^ levels and gastric Na^99m^TcO4 uptake disappeared. There is a slight increase in the Ca^2+^ level, whereas no upregulation of the gastric function was observed (data not shown).

In the current study, EA at acupoints of the stomach meridian in rats showed that EA could upregulate gastric function, which is thought to be correlated with extracellular ions concentrations changes at the acupoints. Further rigorous experimental studies to examine their correlations need to be performed.
